# A Health ‘Kuznets’ Curve’? Cross-Sectional and Longitudinal Evidence on Concentration Indices’

**DOI:** 10.1007/s11205-017-1558-8

**Published:** 2017-01-10

**Authors:** Joan Costa-Font, Cristina Hernandez-Quevedo, Azusa Sato

**Affiliations:** 1 Department of Social Policy, London School of Economics, Houghton Street, London, WC2A 2AE UK; 2 LSE Health, London School of Economics, London, UK

**Keywords:** Concentration indices, Self-reported health, Health inequalities, Kuznets’ curve, Income related health inequalities, I18, O1, I3

## Abstract

The distribution of income related health inequalities appears to exhibit changing patterns when both developing countries and developed countries are examined. This paper tests for the existence of a health Kuznets’ curve; that is, an inverse U-shape pattern between economic developments (as measured by GDP per capita) and income-related health inequalities (as measured by concentration indices). We draw upon both cross sectional (the World Health Survey) and a long longitudinal (the European Community Household Panel survey) dataset. Our results suggest evidence of a health Kuznets’ curve on per capita income. We find a polynomial association where inequalities decline when GDP per capita reaches a magnitude ranging between $26,000 and $38,700. That is, income-related health inequalities rise with GDP per capita, but tail off once a threshold level of economic development has been attained.

## Introduction

The study of income related health inequalities has attracted significant attention in the health policy literature ever since the World Health Organisation employed it as a measurement to compare health system performance (WHS 2002). Estimates from both developed and developing countries show that an individual’s income distribution influences his/her capacity to produce health, which gives rise to income inequalities in health (van Doorslaer et al. 1997; Dolores Montoya Diaz 2002; van Doorslaer and Koolman 2004; Islam et al. 2010). However, we know very little about how such inequalities vary across countries’ income, or economic development.

One can hypothesise that not all countries prefer to ‘prioritize’ the health of everyone in the population, specifically that of poorer individuals. More specifically, changes in macro-determinants such as improvement in the average standard of living and aggregate health transitions exert a very important direct effect on health. Indirectly, for example through the introduction of health insurance, one also sees changes in health outcomes. In considering health inequalities, one could focus on ‘pure inequalities’ in health, which are largely the result of wider socio-economic determinants often outside the scope of health policy action (Schultz 2003), or alternatively, one could focus—as we do here—on income related inequalities in health, which can be influenced by redistribution mechanisms undertaken by governments. Hence, in what follows we explore measures of conditional inequality on health (e.g. concentration indices of self-reported health). Specifically, we test for a health Kuznets’ curve (an inverse U shape association between inequalities and economic development),^1^ which has been widely overlooked in the literature. Indeed, if the level of economic development explains the emergence of health inequalities, then it is a fundamental question to ascertain whether there is an empirical basis for a health Kuznets’ curve. At early stages of industrialisation, inequality is likely to increase but if ‘trickle down’ is successful, inequalities may decrease once the fruits of economic development spread across the entire population.

A classic Kuznets’ curve reflects a quadratic relationship between income inequality and economic development. In the original study, Kuznets (Kuznets 1955) relied on data from only three countries (UK, US, Germany) to test the hypothesis empirically. A long list of studies has followed using both cross-sectional and time series data, but support for a Kuznets’ curve is far from clear-cut. While some studies confirm a Kuznets’ curve (Anand and Kanbur 1993; Saith 1983), others find mixed results (Acemoglu et al. 2002; Ravallion 2005). Given the strong association between income and health, one would expect health related income inequalities to exhibit a Kuznets’ curve, but perhaps with significant differences across countries depending on institutional set-up and policy reactions to health inequalities. Some earlier research focusing in developing countries, draws upon the body mass index (BMI) and calorie consumption as an indicator of wellbeing and fails to find evidence of a Kuznets’ curve (Sahn and Younger 2009) (Haddad et al. 1995) as well as obesity (Grecu and Rotthoff 2015a, b).

This paper examines how the most widely used measure of income related health inequality, namely concentration indices of self-reported health, vary with economic development. In particular, we test for a concave relationship between health inequality (measured as income related inequality) and income (measured as GDP per capita). We take advantage of two large and well-known datasets: the Wold Health Survey (WHS) and the European Community Household Panel (ECHP) survey. The former is a cross-sectional database with large heterogeneity in countries’ economic development, and the latter takes advantage of time-series—cross-section heterogeneity (cross-country characteristics driving the relationships). The datasets contain a representative sample population from Europe and other parts of the world. We believe we provide the first worldwide empirical specification of the phenomenon, to document the effects at a given point in time as well as by using a longitudinal perspective. Previous studies have looked at this pattern using cross-sectional data from different surveys.

We intend to advance the understanding of the relationship between income related health inequalities and economic development. We hypothesise some form of negative association between health inequalities and economic development (as measured by per capita GDP in US $). However, its mechanisms are difficult to explain. On the one hand, countries with better health achievement may decide to invest less in health as marginal productive advantages decline after a certain level of income. Evidence on this is given by the seminal Preston curve (Preston 1975), where an association between income and health is identified but flattens at higher levels of development. Hence, once countries exhibit fewer absolute health improvements they would be expected to switch investment to reducing inequalities. Several studies have been carried out to ascertain and measure income-related health inequalities at the country level. However, despite the heterogeneity in study findings, very few studies have examined what underpins such variation. A second mechanism through which to understand the relationship between income and health inequality is the inverse care law (Hart 1971; Victora et al. 2000). Hart (1971) originally hypothesised that any new treatment may initially be taken up by the rich and not the poor, thus generating health care inequalities which are only later resolved as the poor ‘catch up’. Victora et al. (2000) name this phenomenon the ‘inverse care law’: when new interventions are introduced, richer socioeconomic groups tend to benefit first, thus widening the inequality. It is only after a time lag that poorer socioeconomic groups are able to access interventions, eventually lowering inequality. Examples of interventions include: cervical cancer screening, immunisations and primary health care quality improvements. Lyratzopoulos et al. (2010) documents further such a case for cancer survival between 1973 and 2004. They find evidence of an ‘inequality-equality’ lag cycle, primarily due to the rate of diffusion of new treatments among individuals with different socioeconomic status.

Existing studies, which implicitly refer to a health Kuznets’ curve, are fairly limited. Molini et al. (2010) estimate an association between the Human Development Index (HDI) and the concentration index of BMI in developing countries using quadratic specifications. Importantly, they find an inverted-U relationship between inequalities in BMI and HDI for Vietnam. In contrast, Sahn and Younger (2009) found no evidence of a quadratic curve for BMI-inequality. However, the mean-logarithmic-deviation of women’s BMI increased significantly over the entire GDP range.

The remainder of the paper will examine different specifications of the Kuznets’ curve, measures of health, controls and unobserved heterogeneity. The structure of the paper is as follows. Section 2 contains a description of the data, Sect. 3 presents the empirical strategy and Sect. 4 reports the results and the final section concludes.

## Data

### Choice of Datasets

This paper hypothesizes the existence of a Kuznets’ curve for income related health inequalities both on cross-sectional and longitudinal data. In selecting a cross sectional dataset, we opted to use a geographically wide set, namely the World Health Survey (WHS) data. The WHS is the first major worldwide, nationally representative, survey program to monitor critical health outcomes and health systems through the fielding of a valid, reliable, and comparable household survey instrument. Long and short versions are available at both individual and household levels. In the last round (2003) referring to the period 2002–2004, World Health Organisation (WHO) collected data from 70 countries (for all the different world regions of the WHO^2^). This provided a benchmark for future waves. Samples were probabilistically selected. Sampling weights were generated and adjusted for the population distribution with final post-stratification corrections for non-response. For six countries, China, Ghana, India, Mexico, Russia and South Africa, the sample was the same as that of the Study on Global Ageing and Adult Health (SAGE).^3^ The WHS consists of two questionnaires: household-level and individual-level. Among the questions included in the survey there is a substantial amount of comparable self-reported information on personal health of individuals living in high- middle- and low-income countries (Witvliet 2014).

To test for a Kuznets curve using longitudinal data, we draw upon data from the only available survey that contains a large number of cross-country data over time, namely the European Community Household Panel (ECHP) survey. The ECHP users’ database is a standardised annual longitudinal survey, designed and coordinated by the European Commission’s Statistical Office (Eurostat). It provides up to eight waves (1994–2001) of comparable micro-data on living conditions in the pre-enlargement European Union Member States (EU-15). We did consider EU-SILC but given that the dataset has changed the measure of self-reported health and sample of countries over time, we decided to use ECHP to take advantage of the larger time variation.

### Data Manipulation

To calculate income-related inequalities in self-reported health status, we have considered a binary indicator of self-reported health status, together with equivalised household income. The original SAH question asked respondents: “How is your health in general?”, with five possible answers: “very good”, “good”, “fair”, “poor” and “very poor”. SAH has been used extensively in the literature and has been applied to measure the relationship between health and socio-economic status (Adams et al. 2003); the relationship between health and lifestyles (Kenkel 1995); and the measurement of socio-economic inequalities in health (vanDoorslaer et al. 1997). Some interesting results have been found: self-reported health is a powerful predictor of subsequent mortality (Idler and Kasl 1995; Idler and Benyamini 1997), its predictive power does not vary across socioeconomic groups (Burström and Fredlund 2001), and it is a good predictor of subsequent use of medical care (van Doorslaer et al. 2000) and of mortality (van Doorslaer and Gerdtham 2003). We created a binary indicator of ‘very good or good’ self-reported health status. The income variable is real household income, adjusted using Purchasing Power Parities (PPPs) and the Consumer Price Index (CPI). It is equivalised by the OECD modified scale to adjust for household size and composition.

Tables 3 and 4 contain the description of the data sources, as well as a description of how the data was transformed to produce the relevant indices. In addition, we report definitions used to compute the dependent variable (i.e. the concentration index) and controls applied. As expected the cross sectional dataset in Table 3 exhibits lower GDP and higher income related inequality than that of the longitudinal dataset of European countries in Table 4. In addition to contemporary measures, we examine lagged GDP as a possible covariate, but we found that it was significantly associated with GDP when included in the longitudinal dataset (both together and as a separate variable), given that GDP does not change dramatically from 1 year to the next. The 1 year income lag measure did not show different results either in cross sectional data. Similarly, we considered alternative measures of development such as the Human Development Index (HDI), but the close relationship between life expectancy and health would make its identification problematic. Hence, we decided to rely on the traditional Kuznets’ curve specification that employs straightforward GDP.

Inequality is measured using income related concentration indices (CI), which have been extensively used for measuring inequalities and inequities (Wagstaff et al. 1991). The CI is an index that quantifies the degree of socioeconomic-related inequity in a health indicator (Kakwani et al. 1997; Wagstaff 1989, Costa-Font and Hernández-Quevedo 2012, 2015). Different datasets at the individual and household level were therefore merged to ascertain self-rated health and household socioeconomic status, respectively. The CI for each country is computed using the convenient regression formula (Kakwani et al. 1997; O’Donnell et al. 2008), in which a fractional rank variable is created, and have been used before to measure the effect of institutional changes on inequality (Costa-Font and Gil 2009). We correct for cross-cluster correlation as a form of serial correlation that is likely to be present owing to the rank nature of the regressor (Kakwani, et al. 1997). Finally, the measure of self-reported health used has been dichotomised drawing on different cut-off points from a multiple-category indicator which measures ‘good health’ and its absence. This practice helps to avoid the imposition of some scale even though it results in a loss of some information and it's less sensitive to problems of adaptation (Costa-Font and Costa-Font 2009). We have carried out sensitivity analysis re-running the model expanding to one extra category scale and results were consistent and show negligible variation in inequality indices. Finally, in measuring income related health inequalities, gender and age are typically regarded as the unavoidable components, hence we account for them in the regression estimates rather than using subsamples, which would cut the number of observations drastically.

## Testing for a Kuznets Curve

Empirical studies have also used various functional forms to test the Kuznets’ hypothesis. Some regress inequality measures on per capita income and its inverse. However, in health care, the efficiency-equity trade-off, or the change in the association between health inequalities and economic development, might not only involve socioeconomic development or per capita GDP. Additionally, one can imagine a similar association with regards to health development as per the inverse care law (Hart 1971; Victora et al. 2000), however, the underlying mechanisms are less straightforward, as while economic development is measurable and observable, health development is not equally measurable and well identified to ground generalised policy making trade-offs at country level.

Hence, our strategy has been to estimate a variety of specifications drawing from the simplest quadratic specification, in which coefficients are straightforward to interpret and any inverted U-curve would be easily identifiable:

1 $$CI_{it} = \beta_{0} + \beta_{1} y_{it} + \beta_{2} y_{it}^{2} + \beta_{3} z_{it} + \mu_{t} + \varepsilon_{it}$$

CI refers to concentration index estimates of the two separate measures of health (self-reported health); *y*_*it*_ refers to measures of economic development (e.g., GDP) which are hypothesised to follow a quadratic relationship (y_it_^2^); and *z*_*it*_ relates to other variables which influence health inequalities such as the demographic composition of the population, and *μ*_*t*_ refer to time effects From this specification, it is possible to test whether an inverted-U-shaped relationship is identified such that *β*_1_ > 0 and *β*_2_ < 0. Other possible specifications include *β*_1_ = *β*_2_ = 0 (a flat pattern where no relationship exists) and a monotonic relationship (*β*_1_ > 0 and *β*_2_ = 0). Further to this, the turning point can be obtained. This is the level of per capita GDP (or a health measure if examining health development instead) where inequalities stop increasing and begin to decrease. It is obtained as follows:

2 $$y^{*} = \frac{{ - \beta_{1} }}{{2\beta_{2} }}$$

In addition to measuring the standard trade-off between economic development and health inequalities, an alternative way of thinking about a health Kuznets’ curve is to hypothesize that health inequality might vary with the socioeconomic position of the majority of the population.

To illustrate the potential variation of health inequalities with socioeconomic position, we estimate a Kuznets’ curve on health and health development, using alternative specifications. Anand and Kanbur (1993) suggest a specification that regresses an inequality index on income and its inverse. More precisely:

3 $$CI_{it} = \gamma_{0} + \beta_{1} y_{it} + \gamma_{2} (1/y_{it} )^{{}} + \gamma_{3} z_{it} + \mu_{t} + \varepsilon_{it}$$

The advantage of this specification is that a direct estimate of the turning point can be obtained by taking the square root of the ratio between two regression coefficients. That is:

4 $$y^{*} = \sqrt {\frac{{\gamma_{2} }}{{\gamma_{1} }}}$$

Furthermore, as in Fields and Jakubson (1994) but applied to health, one could expect a similarly shaped Kuznets’ curve across countries but with differing intercepts. If so, one would expect to find significant differences between cross-section and pooled samples, and panel regressions with controls for fixed effects.

Finally, we have estimated different equations, which control for country-specific heterogeneity. Given that the range of the dependent variable varies between −1 and 1, we have accounted for censoring by estimating Tobit models (Greene 2011). The existence of longitudinal data allows us to examine sample changes across different years and hence account for potential compositional effects and more importantly, isolate the effect of country-specific unobserved heterogeneity. Although it is true that economic development will not dramatically vary on a yearly basis, analysis on longitudinal evidence is important for two reasons; First, inequality estimates from longitudinal studies can net out different sources of individual specific unobserved heterogeneity, and hence are arguably more precise. Second, taking advantage of the longitudinal dimension in our regression provides an additional test of how sensitive a Kuznets curve is to the inclusion of time varying covariates.

## Results

**Fig. 1:**
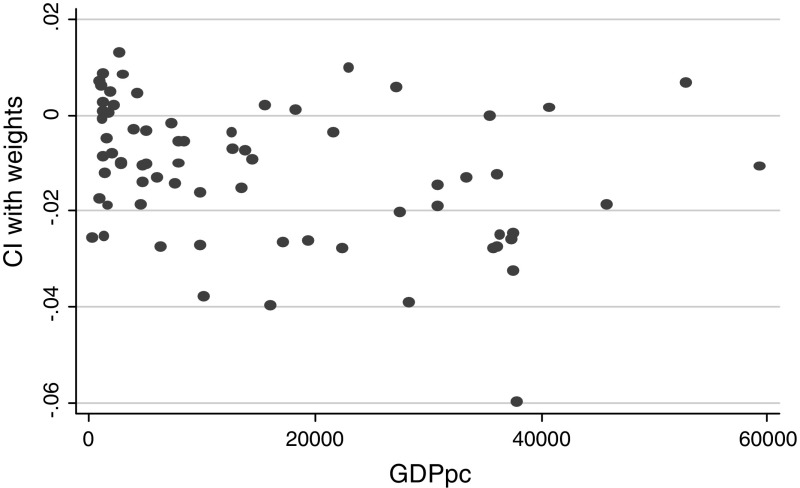
Cross-section health Kuznets’ curve on self-reported health (World Health Survey). *Note*: Income health inequalities are measured using a polynomial Health inequality $(CI_{Hw} )(Y{ - }axis)  and$ Economic Development measures as Gross Domestic Products (GDP) per capita (X-Axis) *Source*: Own calculation on the WHS (2002)

**Fig. 2:**
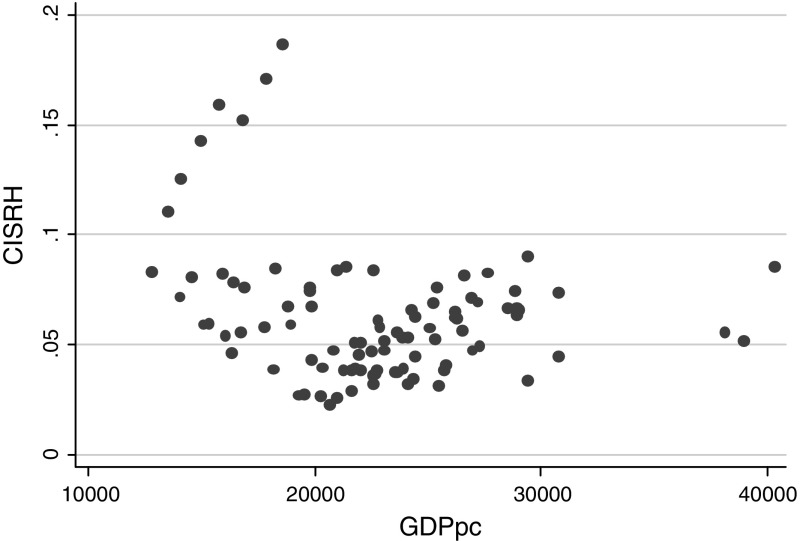
Longitudinal health Kuznets’ curve on self-reported health (European Community Household Panel). *Note*: Income health inequalities are measured using a polynomial Health inequality $(CI_{Hw} ) ({\text{Y}} - {\text{axis}}) and$ Economic Development measures as Gross Domestic Products (GDP) per capita (X-Axis) *Source*: Own calculation on the ECHP (2002)

The simplest results of our strategy can be illustrated in Fig. 1 that shows a plot between the concentration index (CI) estimated using sample weights (although its inclusion did not exert a significant effect on the estimates), for self-reported health and a country’s per capita GDP. It appears as if graphically there is no specific linear relationship. Instead, at first sight, some polynomial association appears to be underpinning the distribution of the data. However, an alternative explanation could be the existence of noise-around-the-mean, which calls for further empirical analysis. When the same association is examined with a sample of European countries in Fig. 2, we also find no clear linear association and again, a specific polynomial association can be seen.

We then proceed with regression analysis drawing upon Ordinary Least Squares (OLS) and then accounting for the censoring of the data through Tobit models. In all specifications, we find conclusive evidence that a quadratic functional form fits the data when the CI is regressed against GDP per capita. The final column of Table 1 provides the estimates of an inverse GDP per capita specification. In addition, we have clustered standard errors by country and corrected standard errors to account for potential heteroscedasticity in the data. Our findings indicate that other study characteristics are mostly insignificant, as well as the development of the health system. Similarly, we find that excluding the three-country observations (that are regarded as outliers) does not change the qualitative conclusion of the results. This is consistent with the view that investment in health care does not appear to reduce health inequalities. Altogether, results suggest evidence of a Kuznets’ curve with a per capita GDP cut–off point ranging from $26,000 to $38,700. In other words, these results suggest that income-related inequalities in self-reported health rise but tail off once a threshold level of economic development has been attained.

**Table 1:** Kuznets’ curves on self-reported health (*CI*_*Hw*_)—cross sectional data from the World Health Survey

	World Health Survey					
	OLS	OLS	Tobit	Tobit	Tobit	Tobit
	Coef. (s.e.)	Coef. (s.e.)	Coef. (s.e.)	Coef. (s.e.)	Coef. (s.e.)	Coef. (s.e.)
*Y*_*i*_	7.67 × 10^−6a^ (2.47 × 10^−6^)	5.17× 10^−6a^ (2.78× 10^−6^)	7.67× 10^−6a^ (2.44× 10^−6^)	5.03E× 10^−6a^ (2.55× 10^−6^)	5.17× 10^−6 a^ (2.66× 10^−6^)	2.84× 10^−6 a^ (1.11× 10^−6^)
*Y*_*i*_^2^	−1.16× 10^−11a^ (4.68× 10^−12^)	−9.87× 10^−12a^ (4.90× 10^−12^)	−1.16× 10^−12a^ (4.62× 10^−12^)	−9.65× 10^−12^)^a^ (4.64× 10^−12^)	−9.87× 10^−12a^ (4.68× 10^−12^)	
1/*Y*_*i*_						1.89302^a^ (1.09907)
Controls	No	Ye	No	Yes	Yes	Yes
Intercept	0.00755^a^ (0.00168)	0.00204 (0.005)	0.00755^a^ (0.00165)	0.005035 (0.00297)	0.002046 (0.0048)	0.0089^a^ (0.00183)
Cluster	Yes	Yes	Yes	Yes	Yes	Yes
Adjusted R^2^	0.15	0.2				
Pseudo R^2^			0.75	0.03	0.03	0.03
Log-likelihood			219.31	221.09	221.26	219.74
Cut-off $Y_{i}$	33,100	26,200	33,100	26,100	26,000	38,700

*Controls* Number of observations, standard error of the concentration indexes

^a^5% level

A further specification using the longitudinal data is made. Again, we find, as reported in Table 2, evidence of a Kuznets’ curve on self-reported health using a generalised least square specification (GLS) given the longitudinal dimension of the data. Importantly, the GDP cut-off points are very much in line with those found in Table 1, ranging from $30,000 to $35,200.

**Table 2:** Kuznets’ curves on self—reported health $({\text{CI}}_{\text{Hw}} )$—longitudinal data from the European community household panel

	GLS		GLS		Tobit		GLS	
	Coef.	(s.e)	Coef.	(s.e)	Coef.	(s.e)	Coef.	(s.e)
*Y*_*i*_	8.73 × 10^−6^5^a^	(2.98 × 10^−5^)	0.00001^a^	(2.97 × 10^−5^)	0.00001^a^	(2.79 × 10^−5^)	6.36 × 10^−5 a^	(3.00 × 10^−5^)
*Y*_*i*_^2^	−1.24 × 10^−9a^	(6.15 × 10^−10^)	−1.71 × 10^−9 a^	(6.32 × 10^−10^)	−1.62 × 10^−109a^	(5.86 × 10^−10^)	−1.06 × 10^−12^ ^a^	(5.76 × 10^−11^1)
Intercept	−0.06959	(0.035290)	−0.11305	(0.03384)	−0.10514	(0.033370)	4.007049	(3.397992)
R^2^	0.13		0.137				0.67	
Fixed effects	No		Yes		No		Yes	
Pseudo								
Controls	No		No		Yes		Yes	
Log-like								
Cut-off $Y_{i}$	35,200		34,8000		34,600		30,000	

*Controls* Number of observations, standard error of the concentration indexes, and population

^a^5% level

## Discussion

This paper documentes evidence of a health ‘Kuznets’ curve reflecting a non-linear (inverse U-shape) association between income-related health inequalities and economic development (GDP per capita). We have drawn upon estimates for both cross-sectional and longitudinal specifications, namely a sample of heterogeneous world countries as well as a panel of different European countries. Our results consistently suggest evidence of a Kuznets’ curve, invariant to the inclusion of other controls for socio-demographic characteristics and specifications.

One interpretation of these findings is that it is only when countries exceed a certain level of income that they can afford to prioritise and target the health of poorer individuals (as opposed to maximising overall health). Explanations for such effect can be linked to the well-known link between democracy (and redistribution) and GDP. Hence, at higher levels of economic development democracy tends to be more developed (Przeworski 2004) and forces such as trade unions tend to play a more active role, contributing to the redistribution agenda. Another way in which redistribution can take place is through the introduction of universal health coverage, in particular, health insurance schemes, which aim to minimise the financial barriers to access to health care, and play a key role in reducing health inequalities. Insurance expansion also reduces the cost of accessing health care by creating larger risk pools, and encourages access to preventive health care which can lead to significant cost savings in health. Another complementary mechanism may be increased demand for health compared to other goods, as incomes grow. Finally, economic development is typically linked to changes in social values (Inglehart 1987). More specifically, prosperity in modern societies allows people to shift attention towards social concerns, including health needs. All these are potential mechanisms at play which merit specific and further analysis.

One potential limitation of our analysis is that our estimates results from self-assessed health measures, which may vary across the income distribution and with economic prosperity as health expectations change. If income is associated to the ability to identify illness symptoms, then the self-reported health measure will systematically underestimate health inequality (Hernández-Quevedo et al. 2008). Another important remark is that our estimates results from income-related health inequality, rather than pure ‘health inequality’ estimates (Costa-Font and Cowell 2013). Hence, the interpretation of our estimates is narrower than traditional Kuznets’ curve explanations.

## Conclusion

Although the World Health Organisation typically ranks health systems based on their performance on reducing health inequalities, we know relatively little about the mechanisms underpinning the variation in income-related inequalities in health. Most previous research has drawn on empirical estimates from heterogeneous surveys and scant theoretical explanation. This paper contributes to the literature by examining one specific (and theoretically grounded) explanation, namely the existence of a Kuznets’ curve on health. Specifically, we argue that a Kuznets curve emerges when we examine the association between (income-related) health inequalities and economic development. Our empirical evidence which draws both from cross –section and longitudinal data unambiguously suggests that the association between (income related) health inequalities and economic development seem to fit a Kuznets’ curve, and more specifically, that health inequalities increase with economic development up to a turning point of GDP per capita varying between $26,000 and $38,700. In other words, it seems that economic development acts as a stimulus to reduce income related inequalities in health.

The immediate policy implication emanating from our results is that countries aspiring to reduce income-related health inequalities should target interventions that advance the effect of economic development on the distribution of health, such as developing prevention programs and efficient mechanisms to insure for the financial costs of health care among others.

Nonetheless, it is important to note that our results can be driven through pathways including the effect of economic development to help curving inequality (e.g., democratic systems pushing for more redistribution and widespread insurance schemes) which help to curb the further expansion of income-related health inequalities. Another explanation lies in the association of income related dimensions of health inequality rather than health inequality per se. The measurement of pure health inequalities is still being subject to ongoing research (Costa-Font and Cowell 2013) Disentangling the latter should be addressed in future research.

## Appendix

See Tables 3 and 4.

**Table 3:** Descriptive statistics World Health Survey sample N = 70. *Source*: World Health Survey 2003

Variable	Definition	Mean	(s.e)
*Inequality measures*			
*CI*_*H*_	Concentration Index (CI) of self-assessed health	0.014	(0.001)
*Economic development measures*			
*Y*_*i*_	Per capita gross domestic product sample 2003	15,276.86	(1807.07)
*Controls*			
*N*_*i*_	Sample size	9804	(1554)
*POP*_*i*_	Population (thousands)	66,801.4	(25,529.9)

**Table 4:** Descriptive statistics European Union Household Panel Survey sample N = 94. *Source*: ECHP several years

Variable	Definition	Mean	(s.e)
*Inequality measures*			
*CI*_*H*_	Concentration Index (CI) of self-assessed health	0.063	(0.0032)
*Economic development measures*			
*Y*_*i*_	Per capita gross domestic product	22,643.3	(544.45)
	*Controls*		
N	Sample size	5371.36	(238.75)
*POP*_*i*_	Population (thousands)	23,355.44	(2398.08)

Countries included: Germany, Luxembourg, UK, Denmark, the Netherlands, Belgium, France, Ireland, Italy, Greece, Spain, Portugal, Austria, Finland

## Footnotes

1 We do not here cover trying to understand the potential different mechanisms underpinning the association between economic development and ‘pure’ health inequalities but solely focus on examining the statsical association between income inequalities in health and economic development. This is becuacse we recognize that pure inequalities have a much larger set of determinantants than income related health incqualities.

2 The survey includes countries from Africa (19 countries), the Americas (7 countries), Europe (30 countries), Eastern Mediterranean (4 countries), South East Asia (5 countries).

3 Further information on the database is available on the WHO website http://www.who.int/healthinfo/survey/en/.
